# Supplementary material for: Atomic Insights into Ti Doping on the Stability Enhancement of Truncated Octahedron LiMn2O4 Nanoparticles
Source: Nanomaterials (Basel). 2021 Feb 17;11(2):508. doi: 10.3390/nano11020508 (PMC7922770; doi:10.3390/nano11020508)
Supplement: Supplementary file 1 [file nanomaterials-11-00508-s001.pdf]

nanomaterials

MDPI

# Atomic Insights into Ti Doping on the Stability Enhancement of Truncated Octahedron $LiMn_2O_4$ Nanoparticles

Wangqiong Xu [1], Hongkai Li [1], Yonghui Zheng [1], Weibin Lei [1], Zhenguo Wang [1], Yan Cheng [1], Ruijuan Qi[1] *, Hui Peng [1], Hechun Lin [1], Fangyu Yue [1] and Rong Huang [1,2,*]

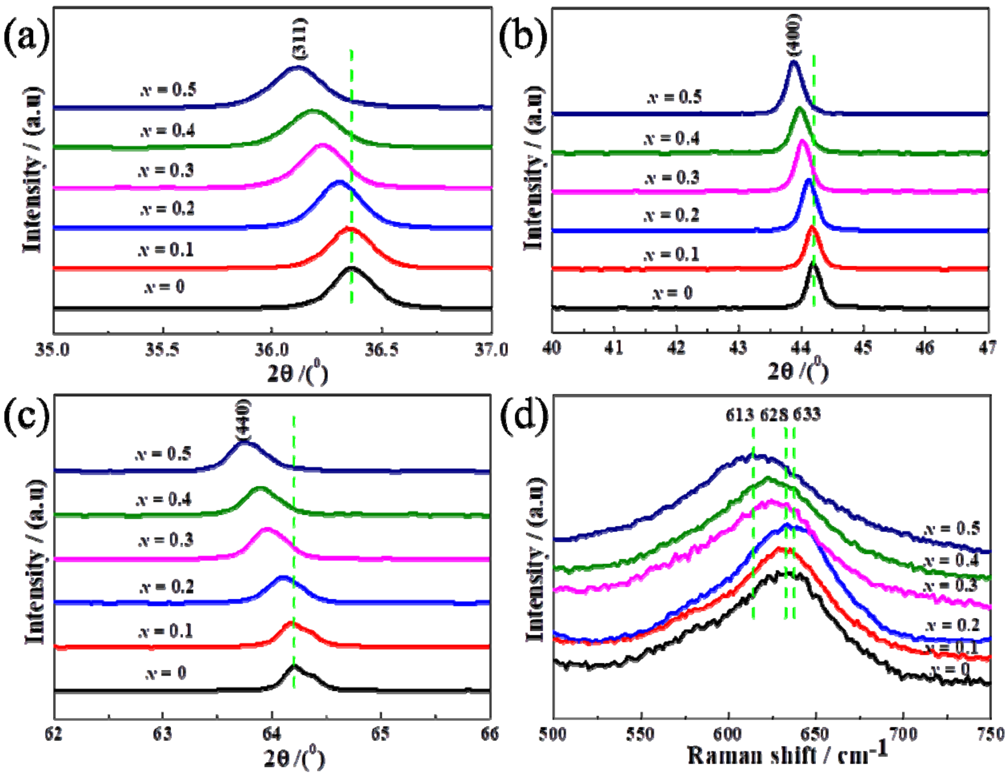


**Figure S1.** Enlarged view of XRD patterns (**a**–**c**) at (311), (400) and (440), and microscopic view of raman spectra at 620 cm$^{-1}$ (**d**).

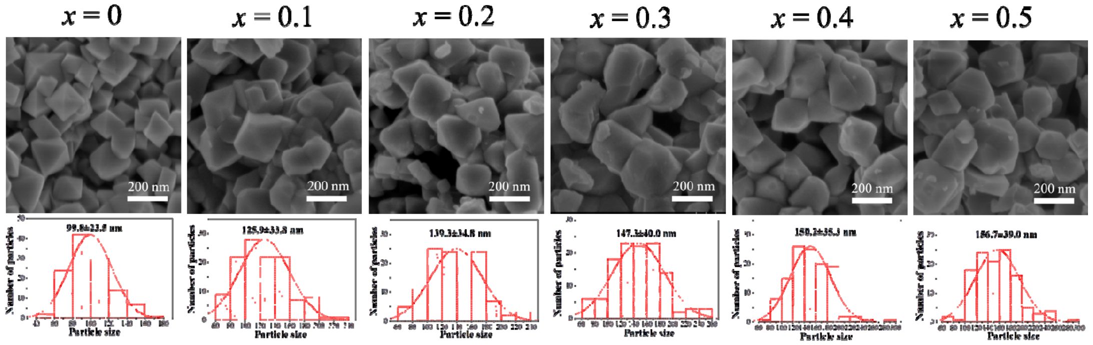


**Figure S2.** SEM images of the $LiTi_xMn_{2-x}O_4$ ($x$ = 0, 0.1, 0.2, 0.3, 0.4, 0.5) at low magnification, and their corresponding to particle size distribution.

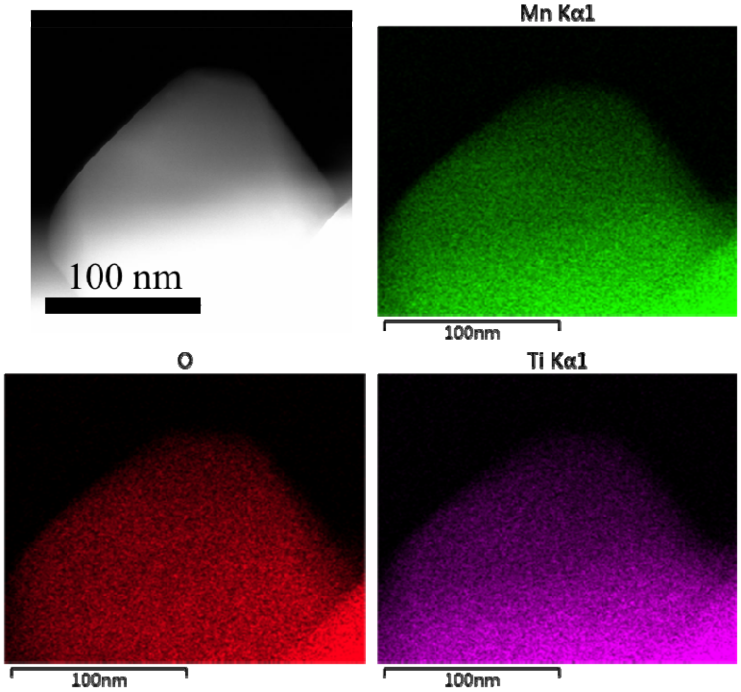


**Figure S3.** STEM image and EDS elemental mappings of the $LiTi_{0.5}Mn_{1.5}O_4$ samples.

The k-factor can be experimentally determined from Equation (1) [1,2]

$$k_{AB} = \frac{\sigma_B(\alpha, \beta, \Delta E)}{\sigma_A(\alpha, \beta, \Delta E)} = \frac{I_B(\alpha, \beta, \Delta E)}{I_A(\alpha, \beta, \Delta E)} X \frac{N_A}{N_B} \quad (1)$$

The quantification of relative chemical concentrations of two elements A and B can be determined via Equation (2) [1,2]

$$\frac{N_A}{N_B} = \frac{I_A(\alpha, \beta, \Delta E)}{I_B(\alpha, \beta, \Delta E)} X \frac{\sigma_B(\alpha, \beta, \Delta E)}{\sigma_A(\alpha, \beta, \Delta E)} \quad (2)$$

| sample / intensity | $LiMn_2O_4$-(111) | $LiTi_{0.5}Mn_{1.5}O_4$-(110) | $LiTi_{0.5}Mn_{1.5}O_4$-(111) | $LiTi_{0.5}Mn_{1.5}O_4$-(001) |
|---|---|---|---|---|
| Mn-$L_{2,3}$ | 4.99784 | 3.63475 | 2.87116 | 3.49802 |
| O-K | 2.94084 | 2.46303 | 2.178 | 2.71725 |
| Mn-$L_{2,3}$/O-K | 1.69946 | 1.47572 | 1.31826 | 1.28734 |
| Mn:O | 0.5 | 0.43386 | 0.38757 | 0.37859 |
| | k=0.294 | | | |

**Figure S4.** Details to quantification the ratio of Mn and O.

**Table S1.** Lattice parameters of $LiTi_xMn_{2-x}O_4$ ($x$ = 0, 0.1, 0.2, 0.3, 0.4, 0.5).

| Sample | Lattice constant a (Å) | Cell volume (Å$^3$) | FWHM of (400) peak (°) | $I_{(311)/(400)}$ |
|---|---|---|---|---|
| $LiMn_2O_4$ | 8.1998 | 551.33 | 0.197 | 0.7728 |
| $LiTi_{0.1}Mn_{1.9}O_4$ | 8.2099 | 553.37 | 0.219 | 0.7794 |
| $LiTi_{0.2}Mn_{1.8}O_4$ | 8.213 | 553.99 | 0.238 | 0.721 |
| $LiTi_{0.3}Mn_{1.7}O_4$ | 8.2302 | 557.49 | 0.238 | 0.674 |
| $LiTi_{0.4}Mn_{1.6}O_4$ | 8.2391 | 559.29 | 0.256 | 0.738 |
| $LiTi_{0.5}Mn_{1.5}O_4$ | 8.2546 | 562.45 | 0.25 | 0.675 |

**Table S2.** Mn $2p_{3/2}$ peak parameters for Mn in $LiMn_2O_4$ sample.

| Peak | B.E. (eV) | FWHM (eV) | Percent (%) |
|---|---|---|---|
| | | $Mn^{3+}$ (total) = 52.72 | |
| $Mn^{3+}$ | 640.2 | 1 | 6.83 |
| $Mn^{3+}$ | 641 | 1 | 14.18 |
| $Mn^{3+}$ | 642.4 | 1 | 21.89 |
| $Mn^{3+}$ | 643 | 1 | 5.81 |
| $Mn^{3+}$ | 645 | 1 | 4.01 |
| | | $Mn^{4+}$ (total) = 47.28 | |
| $Mn^{4+}$ | 641.9 | 1 | 22.55 |
| $Mn^{4+}$ | 643.2 | 1 | 12.88 |
| $Mn^{4+}$ | 643.8 | 1 | 7.32 |
| $Mn^{4+}$ | 644.3 | 1 | 3.51 |
| $Mn^{4+}$ | 646.2 | 1 | 1.02 |

**Table S3.** Mn $2p_{3/2}$ peak parameters for Mn in $LiTi_{0.5}Mn_{1.5}O_4$ sample.

| Peak | B.E. (eV) | FWHM (eV) | Percent (%) |
|---|---|---|---|
| | | $Mn^{3+}$ (total) = 57.86 | |
| $Mn^{3+}$ | 640.4 | 1 | 10.32 |
| $Mn^{3+}$ | 641.1 | 1 | 11.93 |
| $Mn^{3+}$ | 642.2 | 1 | 18.73 |
| $Mn^{3+}$ | 643.1 | 1 | 14.49 |
| $Mn^{3+}$ | 645.2 | 1 | 2.39 |
| | | $Mn^{4+}$ (total) = 42.13 | |
| $Mn^{4+}$ | 641.7 | 1 | 14.58 |
| $Mn^{4+}$ | 642.6 | 1 | 13.51 |
| $Mn^{4+}$ | 643.8 | 1 | 9.11 |
| $Mn^{4+}$ | 644.5 | 1 | 4.19 |
| $Mn^{4+}$ | 642.2 | 1 | 0.74 |

## References

1. Lin, L., Ma, Y.; Wu, J.; Pang, F.; Ge, J.; Sui, S.; Yao, Y.; Qi, R.; Cheng, Y.; Duan, C.-G.; et al. Origin of Photocatalytic Activity in $Ti^{4+}/Ti^{3+}$ Core-Shell Titanium Oxide Nanocrystals. *J. Phys. Chem. C* **2019**, *123*, 20949–20959.
2. Lajaunie, L.; Boucher, F.; Dessapt, R.; Moreau, P. Quantitative Use of Electron Energy-Loss Spectroscopy Mo-M-2,M-3 Edges for the Study of Molybdenum Oxides. *Ultramicroscopy* **2015**, *149*, 1–8.
